# Conversion Surgery via Laparoscopic Gastrectomy and Hepatectomy for Advanced Gastric Neuroendocrine Carcinoma with Extensive Liver Metastases: A Case Report

**DOI:** 10.70352/scrj.cr.24-0110

**Published:** 2025-07-01

**Authors:** Shota Ishii, Kazuhisa Ehara, Hideyuki Kawakami, Naoki Nishie

**Affiliations:** Department of Gastrointestinal Surgery, Saitama Cancer Center, Kita-adachi-gun, Saitama, Japan

**Keywords:** conversion surgery, gastric, neuroendocrine carcinoma, liver metastasis, laparoscopy

## Abstract

**INTRODUCTION:**

Gastric neuroendocrine carcinoma (G-NEC) is a rare but highly aggressive malignancy that often presents with distant metastases and significantly worsens prognosis. Recent studies have suggested that conversion surgery after chemotherapy may improve outcomes in initially unresectable gastric cancers; however, evidence regarding its application in G-NEC remains limited. We report a rare case of advanced G-NEC with multiple liver metastases that was treated with R0 resection through conversion surgery after chemotherapy.

**CASE PRESENTATION:**

A 73-year-old man presented with postprandial heartburn and abdominal pain. Upper gastrointestinal endoscopy revealed a type 3 tumor with submucosal tumor-like elevation. Endoscopic biopsy confirmed G-NEC, and computed tomography revealed lymph node enlargement and seven liver lesions, leading to a diagnosis of T2N1M1 (Stage IVB). The patient was initially treated with etoposide and cisplatin (EP) for unresectable G-NECs. Following 13 courses of chemotherapy, significant tumor reduction was observed, with the disappearance of lymph node metastasis and marked shrinkage of liver metastases. Because all lesions, including liver metastases, were deemed resectable, conversion surgery was performed. The surgical approach consisted of laparoscopic distal gastrectomy with D2 lymph node dissection, laparoscopic left medial hepatic segmentectomy, partial hepatectomy, and cholecystectomy. Pathological examination revealed residual tumor cells at the primary site; however, no viable tumor cells were detected in the lymph nodes or liver resection specimens, indicating a marked response to chemotherapy. R0 resection was confirmed at the final staging of T2N0M0 (Stage IB).

**CONCLUSIONS:**

This case highlights the fact that effective chemotherapy may render initially unresectable G-NECs amenable to curative conversion. Successful R0 resection and a substantial response of liver metastases to EP chemotherapy demonstrated the potential viability of this approach in achieving improved patient outcomes.

## Abbreviations

CE-CT
: contrast-enhanced computed tomography
CR
: complete response
EP
: etoposide and cisplatin
G-NEC
: gastric neuroendocrine carcinoma
IP
: irinotecan and cisplatin
MIS
: minimally invasive surgery
OS
: overall survival

## INTRODUCTION

Gastric neuroendocrine carcinoma (G-NEC) is a rare histological subtype of gastric cancer, accounting for 0.16%–1.48% of all gastric cancers.^1,2)^ G-NEC is known for its high malignancy rate, and distant metastases are observed at the time of diagnosis in 44% of the patients.^1)^ The median survival of patients with metastatic gastroenteropancreatic NEC receiving only the best supportive care is as short as 1 month, and even with optimal treatment, it ranges from 12 to 19 months.^3)^

Recently, conversion surgery following chemotherapy has been observed to improve survival in patients with initially unresectable gastric cancer.^4,5)^ However, surgical resection of NEC metastasis has not yet been recommended,^3)^ and reports on conversion surgery for NEC are scarce. We present a case of advanced G-NEC with multiple liver metastases that was successfully treated with conversion surgery following chemotherapy and achieved curative resection.

## CASE PRESENTATION

**Fig. 1:**
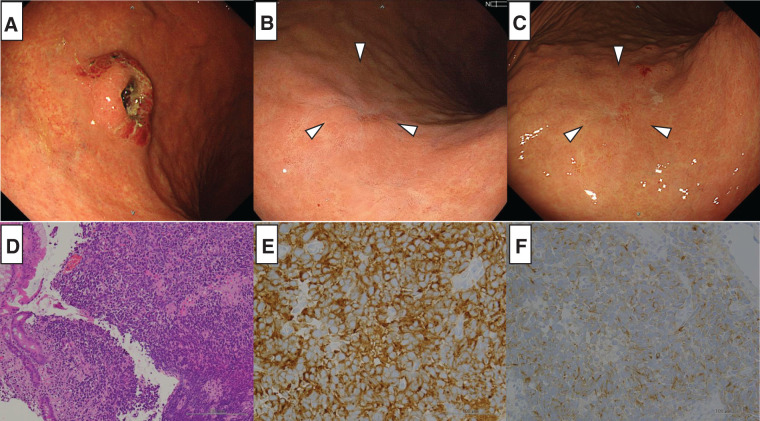
Findings of upper gastrointestinal endoscopy and histological examination from biopsy. (**A**) Before chemotherapy, upper gastrointestinal endoscopy revealed a 20 mm type 3 tumor with a submucosal tumor-like elevation on the greater curvature of the upper body of the stomach. (**B**) After the 6th course of chemotherapy, the tumor markedly shrunk and showed signs of scarring (white arrowheads). (**C**) After the 13th course of chemotherapy, the tumor shrinkage was maintained, and the scarring appearance persisted (white arrowheads). (**D**) Histological findings from the biopsies conducted before chemotherapy. Hematoxylin and eosin staining revealed the diffuse proliferation of cells with a high nuclear/cytoplasmic ratio and naked nuclei. The nuclei varied in size, and the chromatin increased. (**E**) Immunohistochemical analysis before chemotherapy showing positive staining for synaptophysin. (**F**) Immunohistochemical analysis before chemotherapy showing focally positive staining for chromogranin A.

**Fig. 2:**
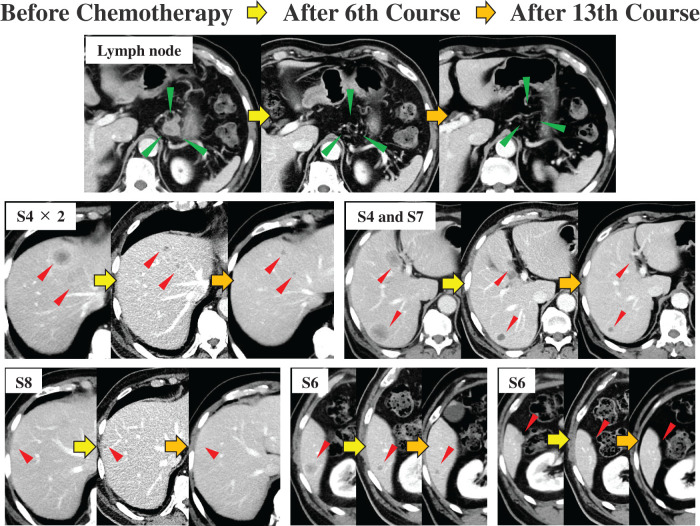
CE-CT findings before, during, and after chemotherapy. Before chemotherapy, a 27 mm mass was observed adjacent to the lesser curvature of the stomach and was identified as a metastatic lymph node (green arrowheads). Multiple liver metastases were also detected (red arrowheads), with three lesions in segment 4 (S4), two in S6, and one lesion each in S7 and S8. After the 6th course of chemotherapy, the metastatic lymph node exhibited marked shrinkage and were almost undetectable. The liver lesions also showed significant shrinkage. The shrinkage of each lesion was maintained even after the 13th course of chemotherapy. All lesions were completely resected during conversion surgery. CE-CT, contrast-enhanced computed tomography

A 73-year-old man presented at another hospital with postprandial heartburn and abdominal pain. He had a 13-year history of hypertension and diabetes mellitus that was managed with antihypertensive medication and oral hypoglycemic agents. Upper gastrointestinal endoscopy at our hospital revealed a 20 mm type 3 tumor with a submucosal tumor-like elevation on the greater curvature of the upper stomach (**Fig. 1A**), prompting an endoscopic biopsy. Histological examination revealed a diffuse proliferation of cells with a high nuclear/cytoplasmic ratio and naked nuclei (**Fig. 1D**). On immunohistochemical staining, the tumor cells were diffusely positive for synaptophysin (**Fig. 1E**) and focally positive for chromogranin A (**Fig. 1F**). These findings were sufficient to establish a diagnosis of NEC. Contrast-enhanced computed tomography (CE-CT) did not detect a gastric lesion, but identified a 27 mm enlarged lymph node on the lesser curvature, suggestive of metastasis. Additionally, seven lesions were found in segment 4 (S4) and S6–S8 of the liver and were diagnosed as multiple liver metastases (**Fig. 2**). The serum tumor marker CEA was elevated at 40.5 ng/mL, while CA19-9 remained within the normal range at 15 U/mL. The tumor was classified as T2N1M1 (Stage IVB) based on TNM classification.

Based on these findings, systemic chemotherapy was initiated using an etoposide and cisplatin (EP) regimen, consisting of 145 mg cisplatin on day 1 and 180 mg etoposide on days 1–3, administered once a month per course. During the treatment, the patient developed grade 4 neutropenia, grade 3 thrombocytopenia, and grade 3 peripheral neuropathy. Consequently, the doses were reduced to 115 mg cisplatin and 145 mg etoposide, and 13 courses were completed.

A follow-up upper gastrointestinal endoscopy after the 6th course of chemotherapy revealed significant tumor shrinkage with scarring (**Fig. 1B**), and this response was maintained through the 13th course (**Fig. 1C**), although histological examination of the biopsy specimen confirmed the presence of residual tumor cells. CE-CT revealed a reduction in both the lymph node and multiple liver metastases (**Fig. 2**), with no new metastatic lesions observed. Additionally, serum CEA decreased to an undetectable level following chemotherapy, while CA19-9 remained unchanged at 10 U/mL. Chemotherapy effectively downstaged the tumor to T1bN0M1 (Stage IVB). Given the marked reduction in liver metastases observed after the 6th course of chemotherapy and the sustained response through the 13th course, these lesions were considered resectable. Consequently, a decision was made to proceed with conversion surgery, including resection of the liver metastases.

**Fig. 3:**
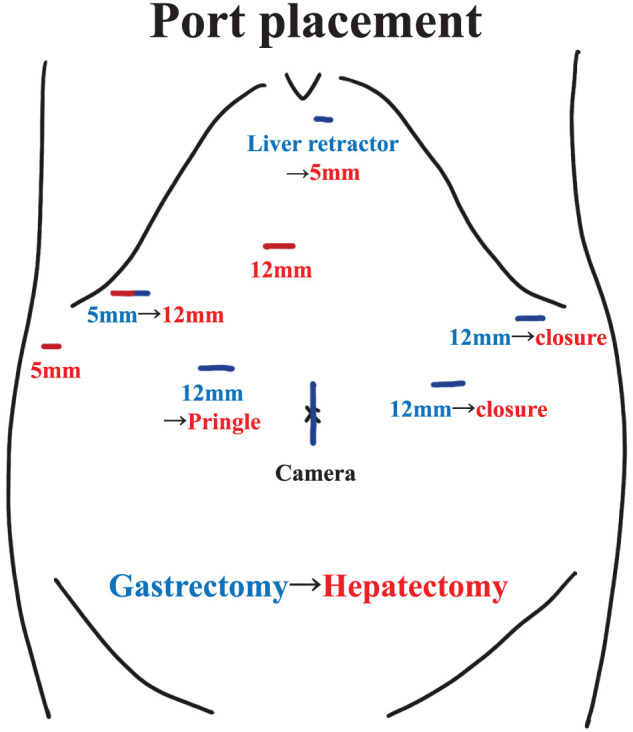
Port placement during conversion surgery. An initial 3 cm skin incision was made around the umbilicus, through which a wound retractor was placed and used as the camera port. Gastrectomy was performed using a five-port setup arranged in an inverted trapezoid configuration, with the addition of a liver retractor positioned in the epigastric region. The ports used during gastrectomy are indicated by blue lines. For the hepatectomy, additional ports (red lines) were inserted while reusing the existing ports from the gastrectomy.

**Fig. 4:**
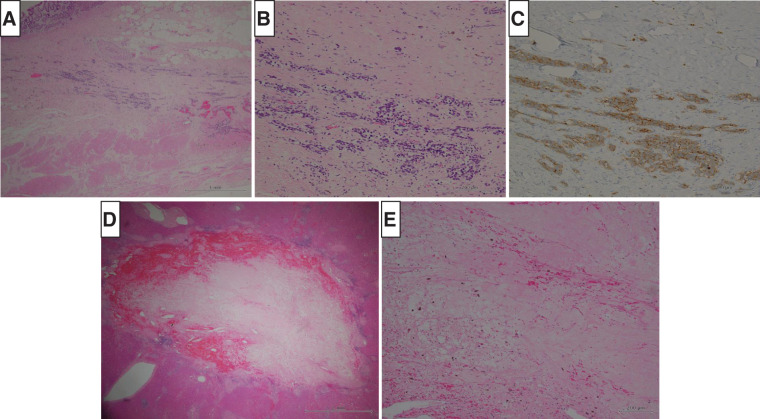
Findings of histological examination from the resected specimens. (**A**, **B**) (high magnification) Hematoxylin and Eosin staining of the stomach. (**C**) Immunohistochemistry (synaptophysin) of the stomach. Residual tumor cells positive for synaptophysin were also observed. The tumor cells had invaded the muscularis propria (MP), and the depth of invasion was diagnosed as T2. The effect of chemotherapy was assessed as grade 2. (**D**, **E**) (high magnification) Hematoxylin and Eosin staining of one of the liver specimens. The lesion had completely transformed into a fibrotic tissue, and no residual tumor cells were observed. The effect of chemotherapy was graded as grade 3.

The patient underwent laparoscopic distal gastrectomy with D2 lymph node dissection and Billroth-II reconstruction, along with laparoscopic hepatectomy and cholecystectomy. The patient was first positioned supine with the legs apart and in a slight reverse Trendelenburg position. The gastrectomy and reconstruction were completed laparoscopically as planned, with the Billroth-II method performed via the antecolic route. The patient was then repositioned into the left semi-lateral decubitus position to proceed with the hepatic resection. Port placement was performed as shown in **Fig. 3**. Intraoperative ultrasonography with Sonazoid contrast enhancement enabled the identification of all seven lesions as hyperechoic nodules. Hepatic resection was performed under vascular inflow occlusion using the Pringle maneuver, after isolating the proper hepatic artery and clamping it with vascular clips, while securing the remaining hepatoduodenal ligament en bloc. Left medial hepatic segmentectomy and cholecystectomy were first performed, followed by mobilization of the right liver. Partial hepatectomy of S7, S8, and two separate sites in S6 were subsequently carried out to achieve complete removal of all lesions. Two drains were placed before concluding the procedure. The entire procedure was successfully completed laparoscopically. The total operating time was 12 h 13 min, with a blood loss of 461 mL. R0 resection, including all identified metastatic sites, was successfully achieved. The postoperative course was uneventful, and the patient was discharged without complications on postoperative day 13. Histological examination of the resected specimens revealed residual tumor cells at the primary gastric site (**Fig. 4A**–**4C**), with grade 2 chemotherapy effects according to the Japanese Classification of Gastric Carcinomas.^6)^ No lymph node metastasis was observed. In the liver, fibrotic changes were noted in S4 and S6–S8, without residual tumor cells, indicating a grade 3 response to chemotherapy (**Fig. 4D**, **4E**). The other lesions were no longer clearly discernible. The final pathological stage was T2N0M0 (Stage IB). The patient is currently undergoing routine follow-up without adjuvant chemotherapy, and remains free of recurrence at 4 months postoperatively.

## DISCUSSION

This case represents one of the few reports of successful conversion surgery for an initially unresectable G-NEC following chemotherapy. Liver metastasis is generally considered a poor prognostic factor for NEC.^7)^ In this case, despite multiple metastases across both liver lobes, effective chemotherapy allowed aggressive surgical intervention and achieved R0 resection. Notably, resected liver metastases showed a significant pathological response to chemotherapy, highlighting the importance of this therapeutic approach.

Conversion surgery is defined as surgical treatment aimed at achieving R0 resection after chemotherapy for tumors initially deemed unresectable or marginally resectable for technical and/or oncological reasons.^8)^ Previous studies have reported that conversion surgery for Stage IV gastric cancer can result in better outcomes than for non-resectable cases.^4,5,9)^ Crucially, achieving R0 resection has been associated with significantly improved prognosis.^4,5,10,11)^ In a large multicenter international retrospective cohort study by Yoshida et al.^8)^ (CONVO-GC-1), the median survival time was significantly higher for patients who achieved R0 resection through conversion surgery at 56.6 months, compared with 25.8 months for R1 resection and 21.7 months for R2 resection. Conversion surgery for unresectable gastric cancer should be considered when the tumor reaches its optimal response to chemotherapy,^12)^ at which point an R0 resection is deemed achievable. This approach is equally applicable to G-NECs, in which achieving R0 resection is considered crucial for improving outcomes. However, the optimal duration of chemotherapy for G-NEC before considering conversion surgery remains unclear due to limited data. In our case, significant tumor shrinkage was observed after 6 courses and maintained through 13 courses. Therefore, conversion surgery was planned at that point when R0 resection appeared feasible.

Historically, open surgery has been the standard approach for conversion surgery. However, in recent years, the effectiveness of minimally invasive surgery (MIS), including laparoscopic and robotic gastrectomy, has been highlighted.^13)^ Recent studies have shown that laparoscopic distal gastrectomy for locally advanced gastric cancer is comparable to open surgery, not only in terms of safety but also in terms of the 5-year survival outcomes.^14)^ Furthermore, Yamamoto et al.^15)^ conducted a retrospective study involving 94 cases of conversion surgery and reported that the MIS group had a significantly lower rate of postoperative complications and achieved better outcomes in terms of recurrence-free survival and overall survival (OS). In the near future, the indications for MIS in conversion surgery are likely to become more common. In this case, a complete laparoscopic approach including liver resection was performed. Reports on MIS involving liver resection^16)^ remain limited, emphasizing the need for further accumulation of cases and assessment of long-term outcomes.

The standard treatment for unresectable G-NEC remains undefined, but EP and IP (irinotecan and cisplatin) regimens are widely used.^1,17)^ A randomized controlled trial conducted across 50 institutions in Japan evaluated the efficacy of EP and IP regimens in gastroenteropancreatic NEC, revealing median OS of 12.5 months for EP and 10.9 months for IP, with no statistically significant difference observed.^18)^ The response rate was 54.5% for EP and 52.5% for IP; however, complete response (CR) was rare and observed in only one patient in the IP group. In this case, EP chemotherapy led to a grade 3 response in liver metastases, achieving CR-like results, as no cancer cells were found in any of the liver resection specimens. Although both regimens are currently considered effective, this case highlights the remarkable efficacy of EP chemotherapy, even in patients with multiple liver metastases, suggesting that it may also be beneficial for similar patients.

In this case, the tumor was ultimately downstaged to IB, allowing for R0 resection. Attempting resection at the appropriate time for patients with good chemotherapy sensitivity may offer the potential for long-term survival. While adjuvant therapy after resection is recommended for localized NEC,^2,3)^ there is currently no evidence regarding adjuvant therapy following conversion surgery. In this case, considering the risk of adverse events, we opted for regular follow-up instead. However, the follow-up period was relatively short, necessitating continuous observation. Another limitation of this case is that PET-CT was not performed during the clinical course. PET-CT might have been useful in evaluating the therapeutic response of the liver metastases and in guiding treatment decisions. If FDG uptake had disappeared in the liver lesions, non-surgical observation could have been considered as an alternative to resection. Further accumulation of similar cases is required to establish the long-term outcomes and postoperative treatment strategies for G-NECs following conversion surgery.

## CONCLUSIONS

G-NEC is a rare and aggressive disease, commonly treated with EP or IP chemotherapy in unresectable cases. When chemotherapy results in significant tumor reduction and renders R0 resection feasible, conversion surgery should be considered. This case suggests that EP chemotherapy may be beneficial in facilitating curative resection, even in advanced stages of liver metastases. However, further research is necessary to establish the optimal postoperative management and long-term outcomes for conversion surgery in G-NEC. Continued case accumulation and studies are essential for refining treatment strategies.

## ACKNOWLEDGMENTS

We would like to thank all the individuals and institutions who contributed to this study.

## DECLARATIONS

### Funding

The authors declare that this study was not funded externally.

### Authors’ contributions

SI described and designed the study.

KE supervised the manuscript writing.

The other co-authors collected the data and discussed the contents of the manuscript.

All the authors have read and approved the final version of the manuscript.

### Availability of data and materials

Data sharing is not applicable to this article, as no datasets were generated or analyzed in the current study.

### Ethics approval and consent to participate

All procedures were performed in accordance with the ethical standards of the responsible committee on human experimentation (institutional and national) and the Declaration of Helsinki of 1964 and later versions. Ethical approval was not applicable as this was a case report.

### Consent for publication

We obtained comprehensive written informed consent from the patient for the publication of clinical details and images related to this case, in accordance with ethical guidelines.

### Competing interests

The authors declare that they have no competing interests.
